# Effect of initial periodontal therapy on gingival crevicular fluid cytokine profile in subjects with chronic periodontitis

**DOI:** 10.1002/cre2.61

**Published:** 2017-04-12

**Authors:** A. Zekeridou, C. Giannopoulou, J. Cancela, D. Courvoisier, A. Mombelli

**Affiliations:** ^1^ School of Dental Medicine, Division of Periodontology University of Geneva Rue Barthélemy‐Menn 19 CH‐1205 Geneva Switzerland; ^2^ Division of Rheumatology University Hospitals of Geneva Geneva, Switzerland

**Keywords:** cytokines, gingival crevice fluid, periodontal disease, periodontal treatment

## Abstract

Cytokines are thought to play an important role in the pathogenesis of periodontal disease. Because periodontal disease is known for its inhomogeneous distribution within the dentition, it is unclear to what extent the detection of various cytokines at different sites correlates with presence of disease. We evaluated whether levels of 12 cytokines in gingival crevicular fluid (GCF) discriminated periodontally diseased sites from healthy ones, or periodontally diseased persons from healthy ones, and assessed the impact of nonsurgical periodontal therapy on these readings. This study included 20 periodontally healthy persons (H) and 24 patients with chronic periodontitis (P). In every participant, we measured the plaque index, gingival index, probing pocket depth (PD), bleeding on probing, and recession at six sites of every tooth. GCF was collected with Durapore^®^ filter strips from two healthy sites (PD<4 mm; HH) in group H, and from two periodontally diseased sites (PD≥5 mm; PP) and two periodontally healthy sites (PD≤3 mm; PH) in group P. The periodontally diseased participants underwent comprehensive nonsurgical periodontal therapy including deep scaling and root planing under local anesthesia. In these participants, GCF samples were again collected at the same sites 1 and 3 months after therapy. Twelve cytokines (il‐1β, il‐1ra, il‐6, il‐8, il‐17, b‐fgf, g‐csf, gm‐csf, ifn‐γ, mip‐1β, vegf, and tnf‐α) were assessed using the Bio‐Plex suspension array system. Mean plaque index, gingival index, bleeding on probing, PD, and recession were significantly different between groups H and P. Differences between PP and PH sites were not significant for any of the cytokines. Il‐1ra, il‐6, il‐17, b‐fgf, gm‐csf, mip‐1β, and tnf‐α differed significantly between HH sites and both PH and PP sites, whereas il‐8 was significantly higher only at PP sites. Periodontal treatment increased gm‐csf and decreased il‐1ra levels in PP sites. Il‐1ra, il‐6, il‐8, il‐17, b‐fgf, gm‐csf, mip‐1β, and tnf‐α identified patients with chronic periodontitis, rather than diseased sites, suggesting a generalized inflammatory state that is not limited to clinically diseased sites only.

## 1 INTRODUCTION

Periodontitis is a multifactorial inflammatory disease that destroys the tissues anchoring the teeth in the jaws. The clinical diagnosis is established based on the presence of periodontal pockets and signs for loss of periodontal tissue and bone. However, such assessments reflect the accumulated damage caused by previous episodes, rather than providing information about the current activity of the disease. As periodontitis is thought to be induced by bacteria that activate the host's innate and eventually adaptive immune systems (Kinane, 2001), levels of biomarkers involved in interactions between bacteria and the host defense may reflect the activity of current disease and reveal information on the risk for future disease (Buduneli & Kinane, 2011; Salvi & Lang, 2015). Recent reviews have summarized the function of various molecules in the host's immune response during periodontitis and have suggested that levels of some constituents of saliva or gingival crevicular fluid (GCF) correlate with disease activity or the risk for disease progression (Buduneli & Kinane, 2011; Kinney, Morelli, Oh, et al., 2014; Kinney, Ramseier, & Giannobile, 2007; Preshaw & Taylor, 2011; Ramseier, Kinney, Herr, et al., 2009).

Cytokines are low‐molecular weight proteins that are produced by various types of cells such as epithelial cells, fibroblasts, lymphocytes, and others. After microbial invasion, cytokines, that are part of the innate immunity response, initiate and propagate the inflammatory pathways of periodontitis (Cekici, Kantarci, Hasturk, & Van Dyke, 2014). Several studies reported higher GCF levels of interleukin (il)‐1β (Becerik, Ozturk, Atmaca, Atilla, & Emingil, 2012; Gilowski, Wiench, Plocica, & Krzeminski, 2014; Konopka, Pietrzak, & Brzezinska‐Blaszczyk, 2012; Mogi et al., 1999; Orozco, Gemmell, Bickel, & Seymour, 2006; Toyman, Tuter, Kurtis, et al., 2015), interleukin‐6 (il‐6), interleukin‐8 (il‐8), and tumor necrosis factor (tnf)‐α (Becerik et al., 2012; Holzhausen et al., 2010; Konopka et al., 2012; Mogi et al., 1999; Zhang, Chen, Zhu, & Yan, 2016) in periodontitis patients than healthy persons. A recent study found the highest GCF levels of vascular endothelial growth factor (vegf) in periodontitis patients, followed by persons with gingivitis and healthy controls (Padma, Sreedhara, Indeevar, Sarkar, & Kumar, 2014).

Periodontal diseases usually do not affect all teeth of the dentition, and tissue destruction can vary substantially even from site to site at the same tooth. Traditionally, unequal disease distribution has been thought to be a consequence of local differences in the quality of plaque control, that is, failure to remove bacterial deposits at some sites. However, the analysis of microbiological and clinical data from large numbers of samples taken in the same dentition of many patients indicated that microbiological parameters alone were unable to fully explain the distribution patterns of periodontal disease (Mombelli & Meier, 2001). In an effort to better understand the reasons for this, and eventually find a biomarker that could predict disease activity at the level of the site, the concentrations of selected cytokines have been assessed at multiple sites in some patients. In periodontitis patients, il‐1β was found to be higher in deep active lesions compared to healthy gingiva (Fujita, Ito, Sekino, & Numabe, 2012; Gamonal, Acevedo, Bascones, Jorge, & Silva, 2000; Oh, Hirano, Takai, & Ogata, 2015; Reis, Da Costa, Guimaraes, et al., 2014; Thunell, Tymkiw, Johnson, et al., 2010; Toker, Poyraz, & Eren, 2008). The same was reported for interferon (ifn)‐γ, il‐6, il‐8, and tnf‐α (Dutzan, Vernal, Hernandez, et al., 2009; Fujita et al., 2012; Papathanasiou, Teles, Griffin, et al., 2014; Reis et al., 2014). On the contrary, interleukin‐1 receptor antagonist (il‐1ra) was found in lower concentrations in deep pockets compared to shallow ones (Toker et al., 2008). Biomarkers have also been assayed repeatedly at diseased sites before and after periodontal therapy. Initial periodontal treatment decreased GCF levels of il‐1β (de Lima Oliveira, de Faveri, Gursky, et al., 2012; Gamonal et al., 2000; Konopka et al., 2012; Oh et al., 2015; Reis et al., 2014; Thunell et al., 2010; Toker et al., 2008), ifn‐γ, il‐8 and granulocyte macrophage colony‐stimulating factor (gm‐csf) (de Lima Oliveira et al., 2012; Gamonal et al., 2000; Konopka et al., 2012; Thunell et al., 2010; Tsai et al., 2007). For il‐6 there is contradictory evidence with either higher or lower levels after treatment (de Lima Oliveira et al., 2012; Reis et al., 2014; Thunell et al., 2010).

Due to small volume, the number of molecules that can be assayed in specimens of GCF from single sites with conventional biochemical methods is restricted. Using multiplex immunoassay techniques, it is, however, possible to analyze multiple biomarkers in small quantities of liquid. This allows a new approach for studying the roles of inflammatory mediators in periodontal diseases that are thought to function in complex networks (Preshaw & Taylor, 2011). Comparing levels of several biomarkers in GCF from healthy and diseased sites in patients with periodontitis to healthy persons and determining changes over time after periodontal therapy are essential steps to elucidate the roles of various mediators and their interaction.

The purpose of the present study was to simultaneously assess GCF levels of 12‐selected cytokines and to analyze to what extent these molecules discriminate sites with periodontitis from healthy sites in diseased persons, to compare levels in periodontally diseased and healthy individuals, and to monitor longitudinal changes in healthy and diseased sites after initial periodontal treatment.

## 2 MATERIAL AND METHODS

GCF levels of 12 cytokines were assessed at multiple sites in 20 periodontally healthy persons (group H) and in 24 patients with periodontal disease (group P). In the latter, specimens were collected from the same sites before and 1 and 3 months after nonsurgical periodontal therapy. The Ethical Committee of the University Hospitals of Geneva, Geneva, Switzerland, approved the protocol. All participants gave written informed consent. Research was conducted according to the principles outlined in the Declaration of Helsinki on human medical experimentation.

### 2.1 Study population

The participants were recruited between October 2013 and March 2016 from persons aged between 30 and 70 years seeking treatment at the University of Geneva, School of Dental Medicine.

Participants of group H did not have any periodontal sites with probing depth (PD) >4 mm, clinical attachment loss ≥1 mm or radiographic evidence of bone loss. The participants of group P presented at least two teeth with probing PD ≥5 mm, clinical attachment loss ≥1 mm, and radiographic evidence of marginal bone loss. We excluded persons with a history of systemic disease (i.e., diabetes mellitus, cancer, HIV, bone metabolic diseases, disorders that compromise wound healing, systemic inflammatory diseases, history of radiation, or immunosuppressive/modulating therapy), with fever or any other signs of infection (upper respiratory, pulmonary, digestive, or urogenital tract) and those who had taken antibiotics in the previous 2 months.

### 2.2 Clinical procedures

In group P, one trained examiner (AZ) collected GCF from the gingival margin of two periodontally diseased (PP) sites (PD ≥5 mm) and two periodontally healthy (PH) sites (PD ≤3 mm). In group H, she collected GCF from two sites (HH) with PD ≤3 mm. Prior to sample collection, the study sites were isolated from saliva with cotton rolls. Every site was carefully cleaned with a cotton pellet and then gently dried with an aspiration tip. After 2 min, a Durapore membrane strip (2 × 6 mm, 0.22‐μm pore size; Millipore, Bedford, MA, U.S.A.) was placed at the gingival margin to collect GCF for 30 s. The strip was immediately transferred into a microtube and stored at −20 °C until further processing. Samples contaminated with blood were discarded. After GCF sampling, the examiner recorded the plaque index (PI) and gingival index (GI) (Loe, 1967), PD, bleeding on probing (BOP), and recession (REC) at six sites per tooth of every tooth.

The patients with periodontitis (group P) then received comprehensive mechanical, nonsurgical periodontal therapy according to the standard operating procedures of the department (Mombelli, Schmid, Walter, & Wetzel, 2015). All periodontally diseased teeth were treated with thorough scaling and root planning to the depth of the pocket under local anesthesia. Depending on the patient's individual treatment needs, the treatment required two to four sessions within 1 month. During this period and one additional week, the participants rinsed the mouth twice daily with 0.2% chlorhexidine. No other local or systemic medications were used. The examiner saw the patients again 1 and 3 months after treatment. Each time she collected GCF at the same sites as before.

### 2.3 Laboratory procedures

Using a multiplex fluorescent bead‐based immunoassay (Kit M5000HIVRK, BioRad Laboratories, Hercules, CA, USA) and the Bio‐Plex 200 suspension array system (Bio‐Rad laboratories, Hercules, CA, USA), we assayed the following biomarkers in each individual GCF sample:il‐1β, il‐1ra, il‐6, il‐8, interleukin‐17 (il‐17), basic fibroblast growth factor (b‐fgf), granulocyte colony‐stimulating factor (g‐csf), gm‐csf, ifn‐γ, macrophage inflammatory protein 1β (mip‐1β), vegf, and tnf‐α. The specimens were eluted in assay buffer in 96‐well filter plates. Microsphere beads coated with monoclonal antibodies against the 12 markers were added. The GCF specimens, standards, and controls were incubated for 30 min. The plates were washed, and a mixture of biotinylated secondary antibodies was added. After a second round of incubation of 30 min the plates were washed again, and streptavidin conjugated to the fluorescent protein phycoerythrin was added. The plates were washed once more after 10 min to remove the unbound reagents, and assay buffer was added. The suspension array system analyzed a minimum of 100 beads per analyte. For the acquisition and processing of the biochemical data, we used the Bio‐Plex Manager Software 3.0 (BIO‐RAD, Hercules, CA, USA). Cytokine data were reported as pg/ml. A constant (0.1) was added to all readings to remove zero values. The detection limits ranged between 1 and 2.24 pg/ml, excepting il‐1ra and tnf‐α with detection thresholds at 5.63 and 6.63 pg/ml, respectively.

### 2.4 Statistical analysis

Patient averages were computed for PI, GI, PD, BOP, and REC. We used Fisher exact test to examine the association of categorical variables with type of participants. The differences between PP and PH sites within the same individual and the longitudinal changes were analyzed with the Wilcoxon signed‐rank test. The differences between healthy sites in healthy people and healthy and periodontitis sites in patients with periodontitis were analyzed with the Wilcoxon rank‐sum test. The Spearman's rank correlation test was used to detect relationship between PP and PH sites. A difference was considered statistically significant if *p* was <.05. R version 3.1.1 (R Foundation for Statistical Computing, Vienna, Austria) was used for these analyses.

## 3 RESULTS

Thirty persons with chronic periodontitis seeking treatment in the pregraduate or postgraduate clinic of periodontology at the University of Geneva, School of Dental Medicine, were identified as potential participants in group P. Twenty‐four of them could be examined, treated and reexamined after 1 and 3 months according to protocol. The remaining six individuals discontinued their treatment prior to examination for personal or financial reasons. One participant missed the 1‐month posttherapy evaluation due to personal reasons. GCF was collected repeatedly at 48 PP sites and 46 PH sites in this group (one of the 24 patients had no healthy sites), thus the levels of 12 cytokines were assayed in 278 specimens from diseased patients. In addition, GCF was collected in 20 periodontally healthy persons at two sites each, yielding 40 HH specimens for analysis.

The demographics and baseline full mouth parameters of the 24 patients of group P and the 20 health controls are shown in Table 1. All parameters were significantly higher in group P (*p* < .05). Among the P group 66% (16 out of 24) of the patients were diagnosed with severe chronic periodontitis (PD ≥7 mm). Table 2 shows the results of the clinical measurements at the sampled sites. As expected, PP sites presented with significantly higher BOP (*p* < .001) and PD (*p* < .001) than PH sites. There was a significant difference for the GI between the PP and PH sites at all time points (*p* baseline = .009, *p* month one = .001, *p* month three = .03). The GI of PP sites was significantly lower after 1 and 3 months than at baseline (*p* = .01, *p* < .001, respectively). All clinical measurements were significantly higher for the PP and PH sites compared to the HH sites (*p* < .05).

**Table 1:** Demographic data and full mouth clinical parameters at baseline. Data are number of participants (percentage) or mean ± standard deviation

Clinical Parameters	Periodontitis patients (*n*:24)	Healthy participants (*n*:20)	*p* value
Sex			
M	14 (58.3)	6 (30.0)	.08
F	10 (41.7)	14 (70.0)	
Smoking			
Yes	15 (62.5)	19 (95.0)	.12
No	9 (37.5)	1 (5.0)	
Age (years)	51.6 ± 10.3	36.2 ± 41,5	**<.001**
PI (% sites with PI > 0)	77.1 ± 27.5	32.5 ± 33.5	**.003**
GI (% sites with GI > 0)	71.9 ± 25.9	12.5 ± 22.2	**<.001**
BOP (% sites with BOP)	70.8 ± 26.2	10.0 ± 20.5	**<.001**
PD (mm)	5.1 ± 0.9	2.3 ± 0.5	<**.001**
REC (mm)	0.8 ± 0.7	0.3 ± 0.6	**<.001**

*Note.* BOP = bleeding on probing; GI = gingival index; PD = probing pocket depth; PI = plaque index; REC = recession. Values in bold: significant difference between groups (*p* < 0.05).

**Table 2:** Clinical parameters at the sites selected for GCF sampling in periodontitis patients. Data are percentage of sites or mean

Parameters	Baseline	Month 1	*p* 1	Month 3	*p* 2
PI (% sites with PI > 0)					
PP	85.4	71.0	.08	73.6	.30
PH	67.4	52.3	.13	41.3	**.03**
*p* value PP vs PH	.052	.07		**.05**	
GI (% sites with GI > 0)					
PP	95.8	76.1	.01	57.2	**<.001**
PH	45.7	36.4	.29	31.8	.21
*p* value PP vs PH	**.009**	**.001**		.03	
BOP (% sites with BOP)					
PP	93.8				
PH	45.7				
*p* value PP vs PH	**<.001**				
Mean PD (mm)					
PP	7.0				
PH	2.9				
*p* value PP vs PH	**<.001**				

*Notes.* BOP = bleeding on probing; GCF = gingival crevicular fluid; GI = gingival index; PD = probing pocket depth; PI = plaque index; PP, periodontally diseased sites in periodontal patients; PH, periodontally healthy sites in periodontal patients; p1, comparison between baseline and 1 month; p2, comparison between baseline and 3 months. Values in bold: significant difference between groups (*p* < 0.05).

The median levels of 12 cytokines for the different sites at baseline are presented in Table 3. Differences between PP and PH sites were not significant. The levels of il‐1ra, il‐6, il‐17, b‐fgf, gm‐csf, mip‐1β, and tnf‐α differed significantly for both PP and PH sites compared to HH sites (*p <* .05). Only il‐8 differed significantly between PP and HH sites (*p* = 0.03). All biomarkers showed a high correlation of the readings at PP and PH sites at every time point (*p <* .01) (Table 4).

**Table 3:** Median GCF cytokine levels (pg/ml) in specimens from patients with periodontitis at baseline and in healthy participants

Baseline	il‐1β	il‐1ra	il‐6	il‐8	il‐17	b‐fgf	g‐csf	gm‐csf	ifn‐γ	mip‐1β	vegf	tnf‐α
PP	291.1	12123.0	5.3	580.5	25.7	31.6	288.0	45.8	41.5	35.7	458.0	14.0
PH	238.0	11708.8	3.8	681.8	23.0	30.0	399.2	45.3	35.7	27.6	299.0	18.5
*p* PP vs PH	.96	.98	.64	.59	.71	.55	.41	.95	.37	.77	.46	.86
HH	373.6	8059.8	37.8	871.0	38.3	53.8	416.1	105.5	35.3	77.3	312.3	27.5
*p* PP vs HH	.89	**.007**	**.005**	**.03**	**.01**	**.005**	.27	**.02**	.70	**.02**	.12	**.049**
*p* PH vs HH	.97	**.007**	**<.001**	.06	**.004**	**<.001**	.86	**.04**	.51	**.02**	.45	**.03**

*Notes.* b‐fgf = basic fibroblast growth factor; GCF = gingival crevicular fluid; g‐csf = granulocyte colony‐stimulating factor; gm‐csf = granulocyte macrophage colony‐stimulating factor; ifn = interferon; il = interleukin; mip = macrophage inflammatory protein; tnf = tumor necrosis factor; vegf = vascular endothelial growth factor; PP, periodontally diseased sites in periodontal patients; PH, periodontally healthy sites in periodontal patients. Values in bold: significant difference between groups (*p* < 0.05).

**Table 4:** Correlations of cytokines between PH and PP sites at baseline and 1 and 3 months after treatment

	Baseline		1 month		3 months	
il‐1β	0.59	**<0.001**	0.61	**<0.001**	0.67	**<0.001**
il‐1ra	0.61	**<0.001**	0.45	**0.002**	0.56	**<0.001**
il‐6	0.60	**<0.001**	0.76	**<0.001**	0.76	**<0.001**
il‐8	0.55	**<0.001**	0.64	**<0.001**	0.50	**<0.001**
il‐17	0.80	**<0.001**	0.77	**<0.001**	0.67	**<0.001**
b‐fgf	0.79	**<0.001**	0.80	**<0.001**	0.74	**<0.001**
g‐csf	0.49	**<0.001**	0.45	**0.002**	0.49	**<0.001**
gm‐csf	0.81	**<0.001**	0.82	**<0.001**	0.83	**<0.001**
ifn‐γ	0.63	**<0.001**	0.59	**<0.001**	0.52	**<0.001**
mip‐1β	0.75	**<0.001**	0.69	**<0.001**	0.79	**<0.001**
tnf‐α	0.78	**<0.001**	0.72	**<0.001**	0.59	**<0.001**
vegf	0.68	**<0.001**	0.54	**<0.001**	0.36	**0.01**

*Notes.* b‐fgf = basic fibroblast growth factor; g‐csf = granulocyte colony‐stimulating factor; gm‐csf = granulocyte macrophage colony‐stimulating factor; ifn = interferon; il = interleukin; mip = macrophage inflammatory protein; tnf = tumor necrosis factor; vegf = vascular endothelial growth factor; PP, periodontally diseased sites in periodontal patients; PH, periodontally healthy sites in periodontal patients. Values in bold: significant difference between groups (*p* < 0.05).

The longitudinal changes of GCF cytokine levels after periodontal treatment are shown in Table 5. Changes from baseline to month one did not reach a level of statistical significance for any analyte. Il‐1ra decreased significantly in PP and PH sites from baseline to month three (*p* PP *<* .001 and *p* PH = .01) but still remained significantly higher than the levels measured in periodontally healthy participants (*p* < .001). On the other hand, gm‐csf increased significantly from baseline to month three for both PP and PH sites (*p* PP = .003 and *p* PH = .009) and those values were similar to those of the HH sites in healthy persons. Changes in levels of the other cytokines were not significant.

**Table 5:** Time trends of median GCF cytokine levels (pg/ml) in PP and PH sites after periodontal treatment

Cytokines	Baseline	Month 1	*p* 1	Month 3	*p* 2
il‐1β					
PP	291.1	248.3	0.95	449.0	0.60
PH	238.0	542.3	0.90	549.0	0.56
*p* PP vs PH	.96	.90		.87	
il‐1ra					
PP	12123.0	12385.0	0.45	9673.0	**<0.001**
PH	11708.8	11385.0	0.65	9899.0	**0.01**
*p* PP vs PH	.98	.97		.69	
il‐6					
PP	5.3	5.9	0.74	28.0	0.89
PH	3.8	4.4	0.55	22.0	0.16
*p* PP vs PH	.64	.88		.62	
il‐8					
PP	580.5	630.1	0.31	879.0	0.24
PH	681.8	535.5	0.86	928.5	0.28
*p* PP vs PH	.59	.38		.63	
il‐17					
PP	25.7	23.0	0.96	30.5	0.42
PH	23.0	26.8	0.45	30.8	0.14
*p* PP vs PH	.71	.81		.50	
b‐fgf					
PP	31.6	27.9	0.93	40.5	0.51
PH	30.0	30.3	0.41	41.0	0.15
*p* PP vs PH	.55	.87		.96	
g‐csf					
PP	288.0	278.0	0.59	419.9	0.19
PH	399.2	368.5	0.98	516.0	0.21
*p* PP vs PH	.41	.68		.35	
gm‐csf					
PP	45.8	55.5	0.08	125.0	**0.003**
PH	45.3	55.8	0.26	112.0	**0.009**
*p* PP vs PH	.95	.83		.95	
ifn‐γ					
PP	41.5	42.0	0.27	31.0	0.12
PH	35.7	41.2	0.69	37.0	0.50
*p* PP vs PH	.37	.76		.44	
mip‐1β					
PP	35.7	30.0	0.41	52.0	0.35
PH	27.6	34.6	0.99	74.0	0.40
*p* PP vs PH	.77	.69		.55	
vegf					
PP	458.0	440.0	0.98	456.5	0.79
PH	299.0	420.8	0.39	458.0	0.10
*p* PP vs PH	.46	.60		.92	
tnf‐α					
PP	14.0	20.9	0.57	23.5	0.43
PH	18.5	19.1	0.59	24.0	0.31
*p* PP vs PH	.86	.71		.92	

*Notes.* b‐fgf = basic fibroblast growth factor; GCF = gingival crevicular fluid; g‐csf = granulocyte colony‐stimulating factor; gm‐csf = granulocyte macrophage colony‐stimulating factor; ifn = interferon; il = interleukin; mip = macrophage inflammatory protein; tnf = tumor necrosis factor; vegf = vascular endothelial growth factor; PP, periodontally diseased sites in periodontal patients; PH, periodontally healthy sites in periodontal patients; p1, comparison between baseline and 1 month; p2, comparison between baseline and 3 months. Values in bold: significant difference between groups (*p* < 0.05).

## 4 DISCUSSION

Using a high‐throughput multiplex fluorescent bead‐based immunoassay system, we recorded the levels of 12 cytokines in 318 samples of GCF in 44 persons. We found significant differences for eight biomarkers (il‐1ra, il‐6, il‐8, il‐17, b‐fgf, gm‐csf, mip‐1β, and tnf‐α) between samples from patients with untreated chronic periodontitis and healthy persons. The limitation in our comparison is that the healthy participants are not matched for age and gender due to difficulty of recruiting persons with no periodontal problems and same characteristics as the test group (Lopez, Scheutz, Errboe, & Baelum, 2007). Our results though are in line with other studies, reporting higher levels of various biomarkers in the GCF of periodontitis patients compared to healthy persons (Gilowski et al., 2014). In contrast, intraindividual differences between sites with (PP) or without (PH) clinical signs of periodontitis were not significant. Our data corroborate findings of other studies suggesting the presence of a generalized inflammatory state in persons with periodontal disease that is not limited to clinically diseased sites. One study evaluated whether GCF levels of myeloid‐related protein (mrp8/14) and its subunits discriminate periodontitis from healthy sites in patients with chronic periodontitis or diseased from healthy subjects (Andersen, Dessaix, Perneger, & Mombelli, 2010). GCF levels of mrp8/14 did not differ between clinically diseased and healthy sites within patients. However, these markers were elevated in periodontally diseased compared with healthy subjects. Earlier studies indicated a similarity in GCF protein concentration (Gustafsson, Asman, & Bergstrom, 1995) or elastase levels (Jin, Soder, Asman, & Bergstrom, 1995; Murray, Mooney, & Kinane, 1995) in sites with various degrees of disease in the same periodontitis patient. In accordance with our findings, a later study (Teles, Sakellari, Teles, et al., 2010) indicated that sites without clinical signs of disease in diseased subjects showed higher levels of several cytokines than healthy sites in healthy subjects.

Furthermore, we detected strong correlation in the expression of all cytokines between PP and PH sites in the same patient. Using the same technology as the present study, a recent analysis (Cionca, Hashim, Cancela, Giannopoulou, & Mombelli, 2016) showed a correlation in the expression of five biomarkers at zirconia implants and teeth (il‐1ra, il‐8, g‐csf, mip‐1β, and tnf‐α), and of four biomarkers (il‐1ra, il‐8, g‐csf, and mip‐1β) at zirconia and titanium implants in the same person, probably reflecting the existence of the same patient‐specific inflammatory response pattern, as we did. This view does not preclude the possibility of occasional instances of high‐local disease activity as evidenced by high levels of il‐1β in deep pockets of patients with aggressive periodontitis (Toker et al., 2008). The two studies had different protocols and analysis. In the first one, the sampling was done with pooled samples from vestibular and lingual sides of zirconia, titanium implants, and teeth whereas, in the second one, only anterior teeth were selected; and the samples were pooled samples taken from the same site with time interval. In both studies, mean concentrations were used for the comparisons. As mentioned in the introduction, other studies reported differences in cytokine levels related to the clinical conditions of the sampled sites. Interpreting and comparing these outcomes, one should consider differences in sampling protocols and analysis, criteria of inclusion of participants, and disease severity (Preshaw & Taylor, 2011).

In the periodontitis patients, we collected GCF from the same sites before and after therapy. Interestingly, there were significant changes in GCF from both PP and PH sites for il‐1ra and gm‐csf, further reinforcing the notion that GCF samples to a large‐part reflect patterns of inflammatory mediators at the level of the person rather than the site. Periodontal therapy may affect acute‐phase reactants that are produced in tissues remote from the infection. Although lower than at baseline, il‐1ra was still significantly higher 3 months after therapy than in healthy sites of healthy persons. In a similar study, initial periodontal therapy lowered 13 of 16 assayed inflammatory mediators in diseased sites, and three of mediators in clinically healthy sites (Thunell et al., 2010).

A recent study compared the clinical and inflammatory changes occurring around implants and natural teeth during and after a phase of undisturbed plaque accumulation (Meyer et al., 2016). Twelve biomarkers were assessed in peri‐implant crevicular fluid and GCF with the same methods as in this study. The different biomarkers reacted inconsistently to the buildup of bacterial biofilm: While il‐1β increased, il‐8 decreased significantly. Mip‐1β decreased significantly in GCF, but not at implants. Within 3 weeks of resumed good oral hygiene, the levels of all biomarkers, assessed at implants or teeth returned to baseline values. Another study assessed changes of biomarkers in GCF as a result of antimicrobial photodynamic therapy of residual periodontal pockets (Müller Campanile, Giannopoulou, Campanile, Cancela, & Mombelli, 2015). Even though beneficial clinical changes were noted, changes in any of the cytokines in GCF were not significant.

In conclusion, the results of this study reflect the complexity of the host response in periodontal diseases. We could not identify distinct candidate markers in GCF to disclose active disease at the level of the site. Il‐1ra, il‐6, il‐8, il‐17, b‐fgf, gm‐csf, mip‐1β, and tnf‐α identified patients with chronic periodontitis, rather than diseased sites, suggesting a generalized inflammatory state that is not limited to clinically diseased sites.
